# Differential Effect of Amylin on Endothelial-Dependent Vasodilation in Mesenteric Arteries from Control and Insulin Resistant Rats

**DOI:** 10.1371/journal.pone.0120479

**Published:** 2015-03-25

**Authors:** Mariam El Assar, Javier Angulo, Marta Santos-Ruiz, Paola Moreno, Anna Novials, María Luisa Villanueva-Peñacarrillo, Leocadio Rodríguez-Mañas

**Affiliations:** 1 Fundación para la Investigación Biomédica del Hospital Universitario de Getafe, Getafe, Madrid, Spain; 2 Instituto Ramón y Cajal de Investigación Sanitaria (IRYCIS), Hospital Ramón y Cajal, Madrid, Spain; 3 Servicio de Análisis Clínicos del Hospital Universitario de Getafe, Getafe, Madrid, Spain; 4 Digestive Diseases Branch, National Institute of Diabetes and Digestive and Kidney Diseases (NIDDK), National Institutes of Health, Bethesda, Maryland, United States of America; 5 Diabetes and Obesity Research Laboratory, Institut d’Investigacions Biomèdiques August Pi I Sunyer (IDIBAPS), Hospital Clínic de Barcelona, Centro de Investigación Biomédica en Red de Diabetes y Enfermedades Metabólicas Asociadas (CIBERDEM), Barcelona, Spain; 6 Department of Metabolism, Nutrition & Hormones, Instituto de Investigación Sanitaria (IIS)-Fundación Jiménez Díaz, Centro de Investigación Biomédica en Red de Diabetes y Enfermedades Metabólicas Asociadas (CIBERDEM), Madrid, Spain; 7 Servicio de Geriatría del Hospital Universitario de Getafe, Getafe, Madrid, Spain; Kobe University, JAPAN

## Abstract

Insulin resistance (IR) is frequently associated with endothelial dysfunction and has been proposed to play a major role in cardiovascular disease (CVD). On the other hand, amylin has long been related to IR. However the role of amylin in the vascular dysfunction associated to IR is not well addressed. Therefore, the aim of the study was to assess the effect of acute treatment with amylin on endothelium-dependent vasodilation of isolated mesenteric arteries from control (CR) and insulin resistant (IRR) rats and to evaluate the possible mechanisms involved. Five week-old male Wistar rats received 20% D-fructose dissolved in drinking water for 8 weeks and were compared with age-matched CR. Plasmatic levels of glucose, insulin and amylin were measured. Mesenteric microvessels were dissected and mounted in wire myographs to evaluate endothelium-dependent vasodilation to acetylcholine. IRR displayed a significant increase in plasmatic levels of glucose, insulin and amylin and reduced endothelium-dependent relaxation when compared to CR. Acute treatment of mesenteric arteries with r-amylin (40 pM) deteriorated endothelium-dependent responses in CR. Amylin-induced reduction of endothelial responses was unaffected by the H_2_O_2_ scavenger, catalase, but was prevented by the extracellular superoxide scavenger, superoxide dismutase (SOD) or the NADPH oxidase inhibitor (VAS2870). By opposite, amylin failed to further inhibit the impaired relaxation in mesenteric arteries of IRR. SOD, or VAS2870, but not catalase, ameliorated the impairment of endothelium-dependent relaxation in IRR. At concentrations present in insulin resistance conditions, amylin impairs endothelium-dependent vasodilation in mircrovessels from rats with preserved vascular function and low levels of endogenous amylin. In IRR with established endothelial dysfunction and elevated levels of amylin, additional exposure to this peptide has no effect on endothelial vasodilation. Increased superoxide generation through NADPH oxidase activity may be a common link involved in the endothelial dysfunction associated to insulin resistance and to amylin exposure in CR.

## Introduction

Amylin, also named islet amyloid polypeptide (IAPP), is a 37 aminoacids peptide that is co-secreted with insulin by the pancreatic β-cells in response to glucose, free fatty acid and food intake [1,2]. Amylin plays a significant role in regulating energy balance by inhibiting food intake and increasing energy expenditure [3,4]. However, infusion of amylin has been shown to cause peripheral insulin resistance in dogs [5], an observation consistent with the fact that transgenic rats for the human amylin gene display glucose dysregulation and insulin resistance [6]. An association between insulin resistance and amylin is supported by studies showing elevated levels of this hormone in patients with impaired glucose regulation [7] and in insulin resistant elderly women [8]. Furthermore, it has been suggested that the degree of insulin deficiency generally correlates with the degree of amylin deficiency [9].

Impaired endothelial vasodilation is an early event that precedes the clinical manifestations of vascular dysfunction, being the first step to cardiovascular disease (CVD) [10,11]. Insulin resistance has been proposed to play a major role in CVD [12–15]. This is probably related to the impairment of endothelium-dependent vascular function associated with insulin resistance, an alteration documented in different distrcits of human and animal vasculature [16–23]. In fact, induction of insulin resistance by feeding rats with high fructose results in defective endothelium-dependent relaxation in both macro- and microvessels [24,25], providing a well-characterized model of endothelial dysfunction [26]. Although we have previously proposed that amylin may interfere with endothelial vasodilation in rat arteries [27], the role of amylin in vascular dysfunction associated with insulin resistance has not been addressed.

An increase in reactive oxygen species (ROS) has been implicated as an important mechanism contributing to endothelial dysfunction and associated to insulin resistance related in part to reduced nitric oxide bioavailability [28]. ROS are a heterogeneous chemical class that includes radicals such as superoxide anion, and hydroxyl radicals, as well as, non radicals such as hydrogen peroxide (H_2_O_2_) [29]. The major sources of ROS in vascular tissue are membrane-associated NADPH oxidases [30]. However, no data are available concerning the role of oxidative stress in the possible impact of acute amylin on endothelial dysfunction.

The aim of the present work was to analyze the effects of amylin on endothelial vasodilation in mesenteric microvessels from control and insulin resistant rats and to determine the possible mechanisms involved.

## Methods

### Animals

Studies were performed in accordance with the Declaration of Helsinki and with the Guide for the Care and Use of Laboratory Animals, as adopted and promulgated by National Institutes of Health, and were approved by the Ethics Committee for Animal Experimentation of the Hospital Universitario de Getafe. All studies involving animals are reported in accordance with the ARRIVE guidelines for reporting experiments involving animals [31]. Male Wistar rats from Harlan Laboratories (Barcelona, Spain) were used in all studies. Rats were kept on a standard pellet diet, tap water *ad* libitum and housed during the experimental period in a room under 12 hour light/dark cycles.

Fructose-fed rats were used as a well characterized model of insulin-resistance (IRR) [32] that has been previously validated by our group [33]. Fructose (20%) dissolved in drinking water was administered to 5 weeks old rats for 8 weeks, at which time the rats developed endothelial dysfunction, manifested by an impaired relaxation to acetylcholine, and insulin resistance as confirmed by the significant increase in the Homeostasis Model Assessment of Insulin Resistance (HOMA) score when compared to age matched rats [33]. In fact, similar fructose treatment duration and post-natal age for starting the treatment are commonly adopted in studies using this rat model [34–36]. Age-matched rats maintained in the same conditions but not receiving fructose in drinking water were used as controls (CR).

Prior to experimental procedures, overnight-fasted rats were weighed and anesthetized with diazepam (5 mg/kg) and ketamine (90 mg/kg). Then, blood samples were obtained via cardiac puncture and collected in anticoagulant-free tubes. Sera were obtained by centrifugation while mesenteric fat pad including mesenteric vascular tree was removed in block for isolation of mesenteric microvessels.

### Biochemical determinations

Circulating levels of insulin (Mercodia AB, Sweden) and amylin (USCN Life Science Inc, USA) were determined in serum by ELISA, following the manufacturer instructions. Serum glucose concentrations were determined using a colorimetric commercial kit (Biolabo SA, Maizy, France). All samples were assessed in duplicate.

Homeostasis Model Assessment of Insulin Resistance (HOMA-IR) index was calculated as described by Mathews *et al*. (1985) [37] and normalized to the value obtained in control rats.

### Measurement of vascular reactivity in isolated mesenteric arteries

Second order mesenteric branches were mounted on a small vessel Mulvany myograph, connected to a digital recorder (BIOPAC Systems, Inc. Santa Barbara, California, USA) for measuring isometric tension, as previously described [38]. The experiments were performed in Krebs Henseleit Solution, (KHS) which was composed of (mM): NaCl 115, CaCl_2_ 2.5, KCl 4.6, KH_2_PO_4_ 1.2, MgSO_4_.7H_2_O 1.2, NaHCO_3_ 25, glucose 11.1, and Na_2_EDTA 0.03. The solution was bubbled with a mixture of 95% O_2_ and 5% CO_2_ to maintain a pH of 7.4. The segments were subjected to their optimal tension (90% of the tension equivalent to an intramural pressure of 100 mm Hg). After a 30 min equilibration period, vessels were exposed to 125 mM K^+^ (KKHS; equimolar substitution of KCl for NaCl in KHS) to check their functional integrity. After a washout period, segments were contracted with 1–3 μM norepinephrine to obtain 80% of the maximal contraction elicited by KKHS. Relaxations to acetylcholine were subsequently assessed by adding increasing concentrations of the drug at 2-minutes intervals (final bath concentrations 1 nM to 10 μM). In order to evaluate the involvement of ROS generation in endothelial dysfunction related to insulin resistance, the microvascular mesenteric segments were pretreated 30 minutes before ACh administration with the superoxide scavenger, the bovine copper-zinc superoxide dismutase (SOD; 100 U/ml), with the H_2_O_2_ scavenger catalase (600 U/ml) and with the NADPH oxidase inhibitor (VAS 2870; 10 μM). For determining the role of ROS in the impact caused by amylin on endothelial vasodilation, microarteries were preincubated for 20 minutes with r-amylin, which was added 15 minutes after addition of SOD, catalase or VAS 2870 to the organ bath. Experiments were systematically performed in a paired way, with parallel analysis of control and treated segments from the same rat. Mesenteric arteries were obtained from a total number of 14 CR and 20 IRR.

### Chemicals

Drugs used were norepinephrine, acetylcholine chloride, bovine copper zinc CuZn SOD, bovine catalase, VAS 2870 (all obtained from Sigma, St Louis, MO), and synthetic rat amylin (r-amylin) (Bachem, Bubendorf, Switzerland). Drug solutions were made in distilled water, except norepinephrine, which was prepared in saline (0.9% NaCl) containing ascorbic acid (0.01% wt/vol) to avoid oxidation and VAS2870 which was dissolved in dimethylsulfoxide (DMSO; final concentration 0.1%).

### Statistical analysis

Results from numerical variables were expressed as mean±SE. The number of rats (N) and the number of vascular segments (n) used for relaxation curves are indicated in each graph. Complete concentration-response (relaxation) curves were compared by two-factors ANOVA. Values of pD_2_ (which is defined as the negative logarithm of the concentration of ACh required to obtain 50% of maximal relaxation) for each group (CR or IRR) and biochemical data were statistically analyzed by unpaired *t*-test. In all cases, a probability value of less than 5% was considered significant. Data were analyzed using version 20.0 of SPSS software (SPSS Corp, Chigaco, IL).

## Results

### Characterization of insulin resistant model (hyperglycaemia, hyperinsulinemia)

Administration of D-fructose (20%) to the rats for 8 weeks resulted in non-significant overweight and moderate but significant hyperglycemia when compared to control-matched rats (p< 0.05), as indicated in Table 1. A marked hyperinsulinemia was also observed, since serum insulin levels were significantly elevated (more than twofold) above the control in rats with insulin resistance (p< 0.0001) (Table 1). The development of insulin resistance in fructose-fed was confirmed by the significant increase in the HOMA score (7.3 ± 1.2 fold increase; p< 0.0001) when compared to control rats.

**Table 1:** Body weight, fasting glucose and insulin of animals.

	Control rats (N = 15)	Insulin resistant rats (N = 25)
Body weight (g)	343.4 ± 18.60	379.2 ± 9.87
Fasting glucose (mg/dl)	65.7 ± 8.28	91.47 ± 5.39 *
Fasting insulin (μg/l)	3.08 ± 0.37	8.73 ± 1.13 ***

Data are expressed as mean±SE. (N) is the number of rats used.

* p< 0.05

*** p< 0.0001 vs control rats by two-tailed unpaired Student′s *t*-test.

**Fig 1:**
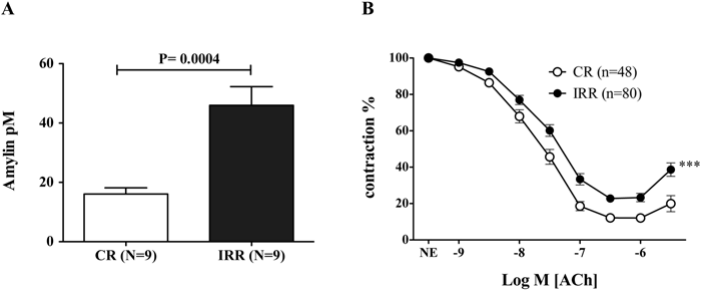
Impaired endothelium-dependent relaxation in insulin resistant rat model and systemic amylin concentration. Panel A shows serum concentrations of amylin (pM) in over-night fasted control (CR) and insulin resistant rats (IRR). Data are expressed as mean±SE. N indicates the number of animals used for determinations. Samples were assessed in duplicate. p = 0.0004 versus CR by unpaired *t*-test. Panel B shows the relaxant response to acetylcholine (ACh; 1 nM to 10 μM) in mesenteric arterial segments derived from CR and IRR. Data are expressed as mean±SE of the remaining contraction induced by norepinephrine (NE). (n) is the number of vascular segments used for each curve. *** p < 0.0001 versus CR by two-factors ANOVA.

In addition, a more than two fold increase in amylin concentration was observed in serum from fructose-fed IR animals versus controls (45.96 ± 6.32 vs 16.09 ± 2.08; p = 0.0004) (Fig. 1A).

### Influence of insulin resistance on endothelium-dependent relaxation and amylin levels

Acetylcholine (ACh; 1 nM to 30 μM) caused endothelium-dependent vasodilatations of rat mesenteric arteries, which were significantly impaired in arteries from IRR when compared to CR (Fig. 1B). The pD_2_ values for ACh were 7.58±0.06 and 7.29±0.06 (p = 0.0035) in CR and IRR respectively.

### Effect of acute amylin treatment on acetylcholine induced relaxation of mesenteric arteries in control and resistant rats

**Fig 2:**
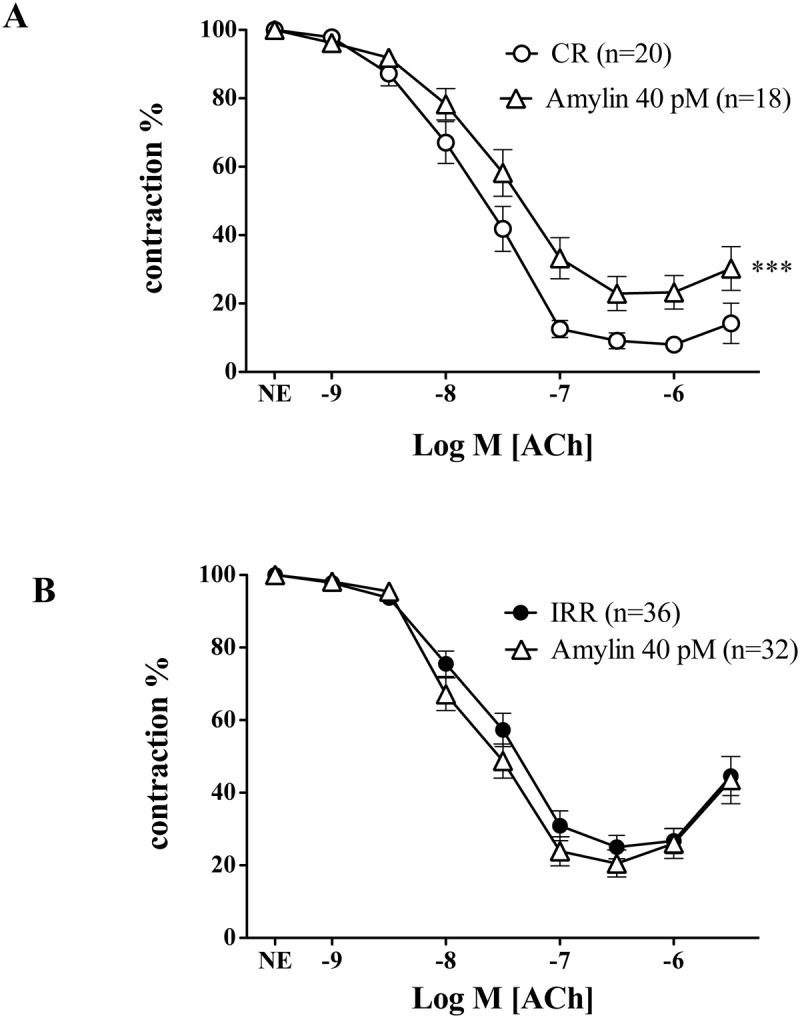
Acute treatment with amylin impairs endothelium-dependent vasodilation only in mesenteric arteries from control rats. Effects of preincubation with r-amylin (40 pM) on endothelium-dependent vasodilation elicited by acetylcholine (ACh; 1 nM to 10 μM) in isolated mesenteric arteries from control rats (CR) (A), and from insulin resistant rats (IRR) (B). Data are expressed as mean±SE of the remaining contraction induced by norepinephrine (NE). n indicates the number of vascular segments used for determinations. *** Indicates p < 0.0001 versus CR by two-factors ANOVA.

As shown in Fig. 2A, treatment of mesenteric arteries of CR with amylin, at a concentration (40 pM) similar to that observed in insulin resistant rats, significantly impaired endothelium-dependent responses induced by ACh. However, in the IRR group that already shows hyperamylinemia and an impaired vascular response to ACh, the preincubation with the same concentration of amylin (40 pM) did not produce a further reduction of endothelium-dependent vasodilation (Fig. 2B).

Preincubation of mesenteric arteries with amylin (40 pM) did not change the basal tone of vascular segments nor altered the contraction to NE in CR or IRR (99.22± 3.56 vs 93.49± 3.71%; 96.39± 3.21% vs 96.46± 3.44% for CR and IRR respectively).

### NADPH oxidase- derived superoxide contributes to endothelial dysfunction either induced by acute amylin treatment in control rats or by insulin resistance

**Fig 3:**
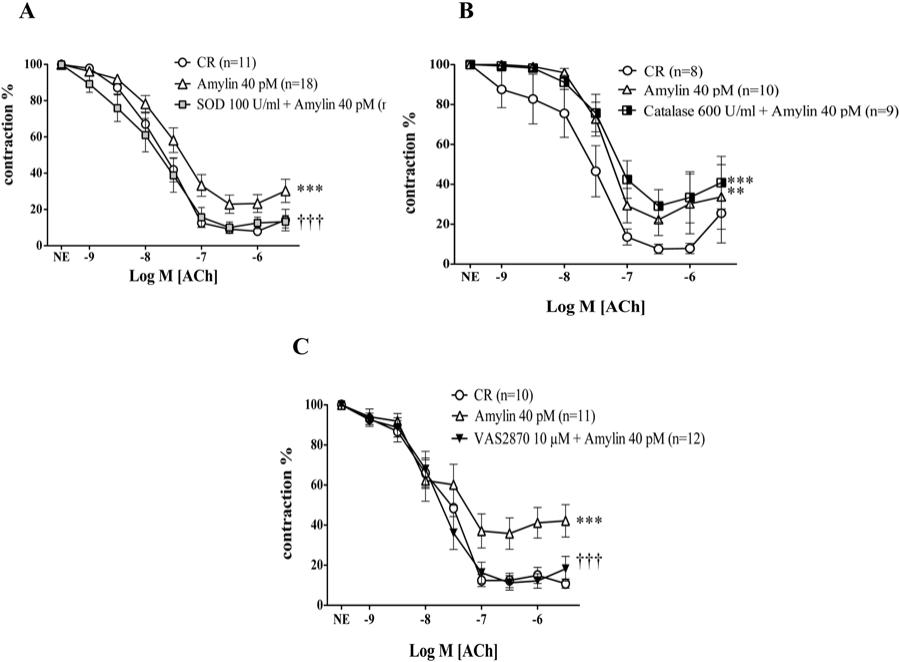
NADPH derived superoxide is involved in the endothelial dysfunction induced by acute amylin treatment in mesenteric arteries of control rats. The impact of the superoxide scavenger, superoxide dismutase (SOD, 100 U/ml) (A), and the hydrogen peroxide (H_2_O_2_) scavenger (catalase, 600 U/ml) (B) on the effects of acute r-amylin treatment (40 pM) on endothelium-dependent relaxation induced by acetylcholine (ACh; 1 nM to 10 μM) in isolated mesenteric arteries from control rats (CR) is shown. The influence of the NADPH oxidase inhibitor, VAS 2870 (10 μM) on relaxant response elicited by acetylcholine (ACh; 1 nM to 10 μM) in mesenteric arteries from CR exposed to acute amylin (Panel C) is also reported. (n) Indicates the number of vascular segments used for determinations. Data are expressed as mean±SE of the remaining contraction induced by norepinephrine (NE). ** Indicates p < 0.001; *** Indicates p < 0.0001 versus CR, ††† indicates p < 0.0001 versus CR plus amylin (40 pM) by two-factors ANOVA test.

To evaluate the participation of superoxide anion generation as a mechanism for amylin inhibitory effect on ACh-induced vasodilations, mesenteric arteries were or not preincubated with superoxide scavenger, superoxide dismutase (SOD, 100 U/ml) for 15 minutes before amylin was added. The presence of SOD completely prevented the inhibitory effects of amylin on ACh induced vasodilation in CR (Fig. 3A). In a similar way, to test the possible implication of the non free radical ROS, H_2_O_2_, microarteries derived from CR were preincubated with catalase (600 U/ml), a scavenger of H_2_O_2_. Catalase was not able to reverse the impairment of ACh-induced vascular responses caused by amylin acute treatment (Fig. 3B). With respect to the possible sources of superoxide production, the role of NADPH oxidase, was analyzed.

Acute treatment of mesenteric arteries of CR with the NADPH oxidase selective inhibitor, VAS 2870 (10 μM) for 15 minutes before amylin was added to the organ bath fully prevented the inhibitory effect of amylin on the relaxations induced by ACh (Fig. 3C).

**Fig 4:**
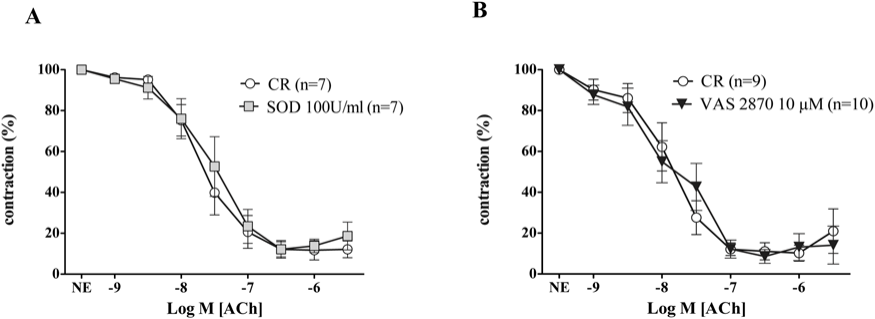
Superoxide scavenging and NADPH inhibition failed to modify endothelium-dependent vasodilation elicited by acetylcholine in control rats in the absence of amylin. The impact of the superoxide scavenger, superoxide dismutase (SOD, 100 U/ml) (A), and the NADPH oxidase inhibitor (VAS 2870, 10 μM) (B) on endothelium-dependent relaxation induced by acetylcholine (ACh; 1 nM to 10 μM) in isolated mesenteric arteries from control rats (CR) is reported. (n) Indicates the number of vascular segments used for determinations. Data are expressed as mean±SE of the remaining contraction induced by norepinephrine (NE).

None of these treatments modified the vasodilation induced by ACh in mesenteric arteries of CR (pD_2_: 7.41± 0.15 vs. 7.56± 0.15; 8.03± 0.20 vs. 7.82± 0.15) for SOD, and VAS 2870, respectively) (Fig. 4A and 4B).

**Fig 5:**
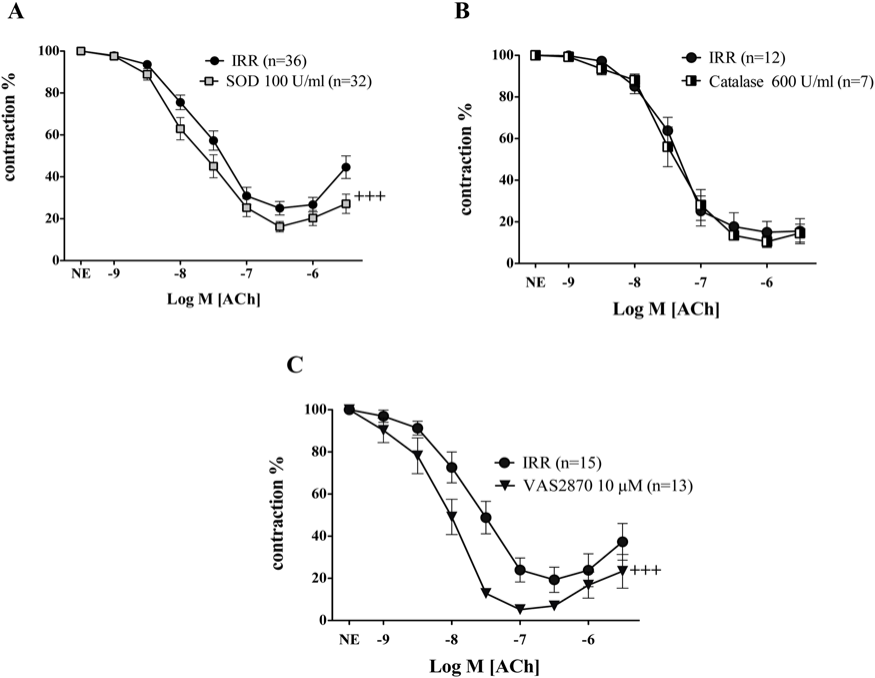
NADPH is the principal source of superoxide anion involved in the endothelial dysfunction associated to insulin resistance. The improving effects of preincubation with the superoxide scavenger, superoxide dismutase (SOD, 100 U/ml) (A) or with the NADPH oxidase inhibitor (VAS 2870; 10 μM) (C) on vasodilation elicited by acetylcholine (ACh; 1 nM to 10 μM) in mesenteric arteries of insulin resistant rats (IRR). Panel B shows the lack of effect obtained when vessels of IRR rats were preincubated with the hydrogen peroxide (H_2_O_2_) scavenger (catalase, 600 U/ml). (n) Indicates the number of vascular segments used for determinations. Data are expressed as mean±SE of the remaining contraction induced by norepinephrine (NE). +++ Indicates p < 0.0001 versus IRR by two-factors ANOVA test.

In mesenteric arteries derived from IRR, while treatment with catalase did not modify ACh-induced vasodilation (Fig. 5B), superoxide scavenging with the extracellular detoxifying enzyme SOD (100 U/ml) significantly improved responses elicited by ACh (Fig. 5A). In microarteries precontracted with NE, the pretreatment with VAS 2870 also ameliorated the endothelium-dependent vasodilation induced by ACh (Fig. 5C). By opposite, the improving effect induced by SOD, or VAS 2870 was not altered by the additional presence of amylin (40 pM) in mesenteric arteries from IRR (data not shown).

## Discussion

The present results suggest that elevated levels of amylin could contribute to the impairment of endothelium-dependent vasodilation observed in insulin resistance through generation of superoxide anions. This is based on the fact that the impairment of endothelial vasodilation in mesenteric microarteries from insulin resistant rats is associated with increased concentration of serum amylin. When similar concentrations of this peptide are added to vessels from control rats, defective endothelium-dependent vasodilation develops but not when added to the vessels from insulin resistant rats. Amylin-induced effects in arteries from control rats are reversed by detoxifying superoxide anions or by inhibiting superoxide production by NADPH oxidase. Improvement of endothelial vasodilation in vessels from insulin resistant rats is accomplished by scavenging superoxide even in the absence of exogenously added amylin.

Fructose-fed rats represent a well- characterized model of insulin resistance [33,26], which is confirmed by the presence of mild hyperglycemia and marked hyperinsulinemia in our experimental setting. Furthermore, HOMA score, commonly used as an index of insulin resistance, was notably increased in 8 week fructose fed animals when compared to control matched rats. We also found more than twofold increase in serum amylin concentrations in insulin resistant rats. This finding is consistent with the increased levels of amylin detected in humans under insulin resistance conditions such as impaired glucose regulation, type 2 diabetes [7] and polycystic ovary syndrome [39]. The alteration of metabolic hormones levels is associated with a significant impairment of endothelium-dependent relaxation in mesenteric arteries from these rats when compared to control, non-insulin resistant, rats. This is in agreement with previous studies reporting reduced endothelium-dependent vasodilation in fructose-fed rats and other rat models of insulin resistance [24,25,40].

To ascertain if high concentrations of amylin could contribute to the endothelial impairment observed in IRR, mesenteric arteries from control rats were exposed to amylin at concentrations (40 pM) similar to those reached in serum from IRR (~46 pM). At such concentration, amylin caused an impairment of endothelial vasodilation, suggesting that the presence of high levels of amylin could account for the defective endothelial vasodilation in microvasculature of IRR. This observation is consistent with previous results showing the capacity of exogenously added amylin to impair endothelium-dependent relaxation of rat aorta and mesenteric arteries [27]. Former reports had suggested a vasodilatory capacity of amylin but these studies were carried out with concentrations of amylin largely above those achievable in plasma, even in insulin resistance, an effect related to its agonistic activity on calcitonin gene-related peptide (CGRP) at such elevated concentrations [41,42], that does not seem to be mediated by NO or the endothelium [43].

The lack of further impairment of endothelium-dependent vasodilation in mesenteric arteries from IRR when exposed to 40 pM amylin supports the hypothesis of a contribution of increased amylin to endothelial impairment in insulin resistance. In other words, if high amylin is interfering with endothelial vasodilation in these animals, the addition of exogenous amylin does not exert additional effects, as it could be expected if a different mechanism was responsible.

It has been proposed that amylin causes cardiac dysfunction in insulin resistance as suggested by the increased deposition of amylin in failing hearts from obese and type 2 diabetic patients [44]. This association is supported by the fact that overexpression of human amylin causes cardiac dysfunction in rats through deposition of amylin oligomers [44]. Although we cannot entirely discard the formation of oligomers of amylin when added to microvessels in our study, this mechanism is not likely responsible for the impairment of endothelial vasodilation driven by amylin since we have used rat amylin that is considered not to be amyloidogenic [45].

The deleterious effect of amylin on endothelial vasodilation of rat mesenteric arteries probably involves generation of superoxide anions by NADPH oxidase, as suggested by the preventive actions of the extracellular superoxide scavenger, SOD and the NADPH oxidase inhibitor, VAS2870. The possible implication of the non free radical ROS, H_2_O_2_, in the impairment of endothelial vasodilation induced by amylin is unlikely since catalase, that breakdowns H_2_O_2_, had no effect on defective vasodilation caused by amylin. Therefore, this observation reinforces our conclusion with respect to superoxide anion generated by NADPH oxidase as the principal culprit of endothelial dysfunction associated to amylin acute treatment. Although this finding has been obtained with non-amyloidogenic rat amylin, some evidences suggest an association between deposition of amyoloidogenic human amylin and increased oxidative stress levels. Indeed, inhibition of amyloid formation prevented the induction of oxidative stress in human islet amyloid polypeptide transgenic mouse islets in culture [46].

It is of importance to point out that there are no data in literature providing the possible mechanisms underlying the defective responses induced by acute amylin treatment in isolated mesenteric arteries derived from animals with preserved endothelial function.

Similarly to the mechanism driven by acute amylin in CR, superoxide generation rather than H_2_O_2_ is involved in the impairment of endothelial vasodilation in IRR since the treatment of mesenteric arteries from these animals with SOD, significantly improved endothelial responses while catalase failed to do so. Previous data in mild pre-diabetic insulin resistant mice proposed that blunted vasorelaxation responses to ACh arise secondary to an increase in endothelial cell production of ROS. Furthermore, the impaired endothelium mediated relaxation of aortic segments in these animals was restored by a SOD mimetic [47].

In vascular tissue, the membrane-associated NADPH oxidase accounts for the majority of superoxide generation favoring the production of ROS [30]. In our experimental model of insulin resistance, VAS 2870 preincubation improved ACh-induced vasodilation in mesenteric arteries of fructose fed rats. Although limited evidences suggests off-target activities by the NADPH oxidase inhibitor, VAS2870 [48], it should be noted that most of scientific evidences point to VAS2870 as displaying consistent specificity to inhibit NADPH oxidase [49,50]. Thus, VAS2870-induced effects are consistent with available data in which an improvement of endothelial dysfunction was observed by specifically targeting NADPH oxidase in insulin resistant mice [51]. Taken as a whole, all these facts suggest that a common mechanism contributes to endothelial dysfunction caused by insulin resistance in mesenteric microvessels and the endothelial dysfunction induced by increasing concentrations of amylin to pathological levels in mesenteric arteries from healthy rats. In fact, defective NO-mediated responses are responsible for the impairment of endothelium-dependent relaxation caused by amylin in aorta of CR [27], a mechanism also involved in the endothelial dysfunction detected in aorta from IRR [33]. This suggests that reduction of NO availability caused by NADPH oxidase stimulation by amylin excess or insulin resistance could compromise endothelial vasodilation in rat vasculature.

In conclusion, amylin impairs vasorelaxant responses in isolated mesenteric arteries only when endothelial function is preserved and endogenous amylin is not elevated. In the presence of insulin resistance, characterized of defective relaxant response to acetylcholine and hyperamylinemia, exogenously added amylin has no effect. One potential mechanism implicated in the endothelial dysfunction associated to amylin increase is related to superoxide generation by means of the NADPH oxidase system a mechanism also contributing to endothelial dysfunction in insulin resistance and hyperamylinemic conditions.
